# Stability of Proton Superoxide and its Superionic Transition Under High Pressure

**DOI:** 10.1002/advs.202415387

**Published:** 2025-01-13

**Authors:** Zifan Wang, Wenge Yang, Duck Young Kim

**Affiliations:** ^1^ Center for High Pressure Science & Technology Advanced Research (HPSTAR) Shanghai 201203 P.R. China; ^2^ Shanghai Key Laboratory of Material Frontiers Research in Extreme Environments (MFree) Shanghai Advanced Research in Physical Sciences (SHARPS) Pudong Shanghai 201203 P. R. China

**Keywords:** high pressure, phase transition, superionicity, superoxides

## Abstract

Under extreme conditions, condensed matters are subject to undergo a phase transition and there have been many attempts to find another form of hydroxide stabilized over H_2_O. Here, using Density Functional Theory (DFT)‐based crystal structure prediction including zero‐point energy, it is that proton superoxide (HO_2_), the lightest superoxide, can be stabilized energetically at high pressure and temperature conditions. HO_2_ is metallic at high pressure, which originates from the 𝜋^*^ orbitals overlap between adjacent superoxide anions (O_2_^−^). By lowering pressure, it undergoes a metal‐to‐insulator transition similar to LiO_2_. Ab initio molecular dynamics (AIMD) calculations reveal that HO_2_ becomes superionic with high electrical conductivity. The possibility of creating hydrogen‐mixed superoxide at lower pressure using a (Li_x_,H_1‐x_)O_2_ hypothetical structure is also proposed. This discovery bridges gaps in superoxide and superionicity, guiding the design of various H‐O compounds under high pressure.

## Graphical abstract

The study predicts the stable proton superoxide (HO_2_) under extreme conditions using Density Functional Theory (DFT)‐based crystal structure prediction. HO_2_ exhibits metallic behavior at high pressure, transitions to an insulator under reduced pressure, and becomes superionic with high electrical conductivity under elevated temperatures. The possibility of creating hydrogen‐lithium mixed superoxides at lower pressures is also explored.

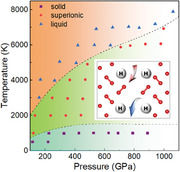

## 1 Introduction

Superoxide is a class of special compounds with peculiar superoxide anions (O_2_^−^) and simple stoichiometry. The superoxide anions O_2_^−^, charged oxygen molecules with an extra electron, play an important role in biology and chemistry and are also the key for a variety of novel physical phenomena.^[^ ^1^ ^]^ O_2_^−^ possesses nine electrons in the 2p molecular orbitals, resulting in a partially filled 𝜋^*^ orbital due to the three electrons in the four‐fold degenerate 𝜋^*^ orbitals. Consequently, alkali superoxides such as *A*O_2_ (*A* = Na, K, Rb, and Cs) exhibit complex and fascinating magnetic properties with couplings between spin, orbital, and lattice, including the Mott insulating behaviors with the strong Hubbard interactions, the antiferromagnetic‐paramagnetic transition, structural transition from the high‐temperature to low‐temperature phase.^[^ ^2^ ^]^

NaO_2_ and KO_2_ are known as key materials in air batteries, where O_2_^−^ ions play a critical role in the electrochemical process. These superoxides were observed to form reversibly and exclusively as solid discharge products under the condition of single‐electron transfer per formula unit, occurring at remarkably low overpotentials. Hartmann et al. demonstrated a Na–O_2_ cell exhibiting high current densities (0.2 mA cm^−2^) and low overpotentials (<200 mV) utilizing stable NaO_2_ as the primary discharge product.^[^ ^3^ ^]^ Similarly, Qin et al. reported a K‐O_2_ battery that employs KO_2_, achieving low round‐trip overpotentials and high coulombic efficiencies.^[^ ^4^ ^]^

LiO_2,_ it is a recently synthesized one in the family of lithium‐ion batteries, which has the great potential to replace lithium peroxide counterpart as the discharge product because of its high conductivity.^[^ ^5^ ^]^ Lithium‐ion batteries attract much attention on metal‐O_2_ battery development because it not only can store an amount of energy in a such small size but has an energy density that is comparable to gasoline.^[^ ^6^ ^]^

Thus, an idea immediately comes out when we make our rounds in the periodic table of elements: does HO_2_ exist? If it does, it would be the lightest superoxide. In addition, its stability against the well‐known H_2_O at extreme conditions may open up another research direction to the study of exoplanets. However, to our best knowledge, few studies paid attention to this possible proton superoxide HO_2_.

Pressure is an effective way to control the physical and chemical properties of structures as a thermodynamic parameter. The increase in pressure can make the volumes and also the distances between atoms greatly decrease, which will cause strong interactions between electrons–electrons, electrons–nucleus, and nucleus–nucleus which further changes the structures and properties of materials. In this way, many unexpected new compounds and new states that cannot exist at ambient conditions will emerge under high pressure. For instance, reactive metal sodium can react with inert helium to form Na_2_He compounds under high pressure.^[^ ^7^ ^]^ Many novel stoichiometries will appear on sodium chloride.^[^ ^8^ ^]^ Discovered iron peroxides FeO_2_ provide us a new perspective on the oxygen cycle inside our Earth.^[^ ^9^ ^]^

Several lithium superoxides have been predicted and synthesized under high pressure including Li_2_O_3_, LiO_2_, and LiO_4_.^[^ ^10^ ^]^ Similarly, this can be a beacon for our exploration of HO_2_. There is high possibility that HO_2_ may exist under high pressure. This is because the enthalpic effect varies between peroxides and superoxides. Due to having fewer electrons and thus a smaller volume than O_2_^2‐^ in peroxides, the O_2_^−^ in superoxides is naturally and favorably formed under high pressure because of the PV term in enthalpy. KO_2_ and NaO_2_ are formed at ambient pressure and they are used to design batteries.^[^ ^3^, ^11^ ^]^ LiO_2_ is a high‐pressure phase and is metastable at ambient pressure.^[^ ^5^, ^10^ ^]^ Similarly, HO_2_ may be able to be stabilized at high pressure.

Furthermore, there are existing studies on the H‐O system under various pressure‐temperature conditions, including water ice and hydrogen clathrates.^[^ ^12^ ^]^ Researchers have discovered several novel stable H‐O compounds under conditions found in the interiors of ice giants, such as H_3_O and H_4_O,^[^ ^13^ ^]^ which could explain some of the enigmatic features of these planets, including their anomalous magnetic fields. However, these compounds are all hydrogen‐rich, and there is limited research on oxygen‐rich compounds under such conditions. Notably, Mao et al. and Chen et al. found new hydroxides from H_2_O and H_2_O_2_/H_2_O mixtures in high‐pressure X‐ray experiments, although the crystal structures were not clearly reported.^[^ ^12^, ^14^ ^]^ Therefore, it is of great significance and necessity to investigate HO_2_ under high‐pressure conditions.

In this study, we have identified stable HO_2_ under extreme pressure using crystal structure predictions. We assessed its stability, calculated its electronic structure, and analyzed it using first‐principles calculations. Ab initio molecular dynamics simulations reveal its superionic behavior. Furthermore, we discussed the implications of creating a hydrogen‐mixed superoxide toward lower pressure within the Li‐O‐H system, which may enhance our understanding of superoxide and superionicity.

## 2 Results and Discussion

### 2.1 Structure and Stability

**Figure 1:**
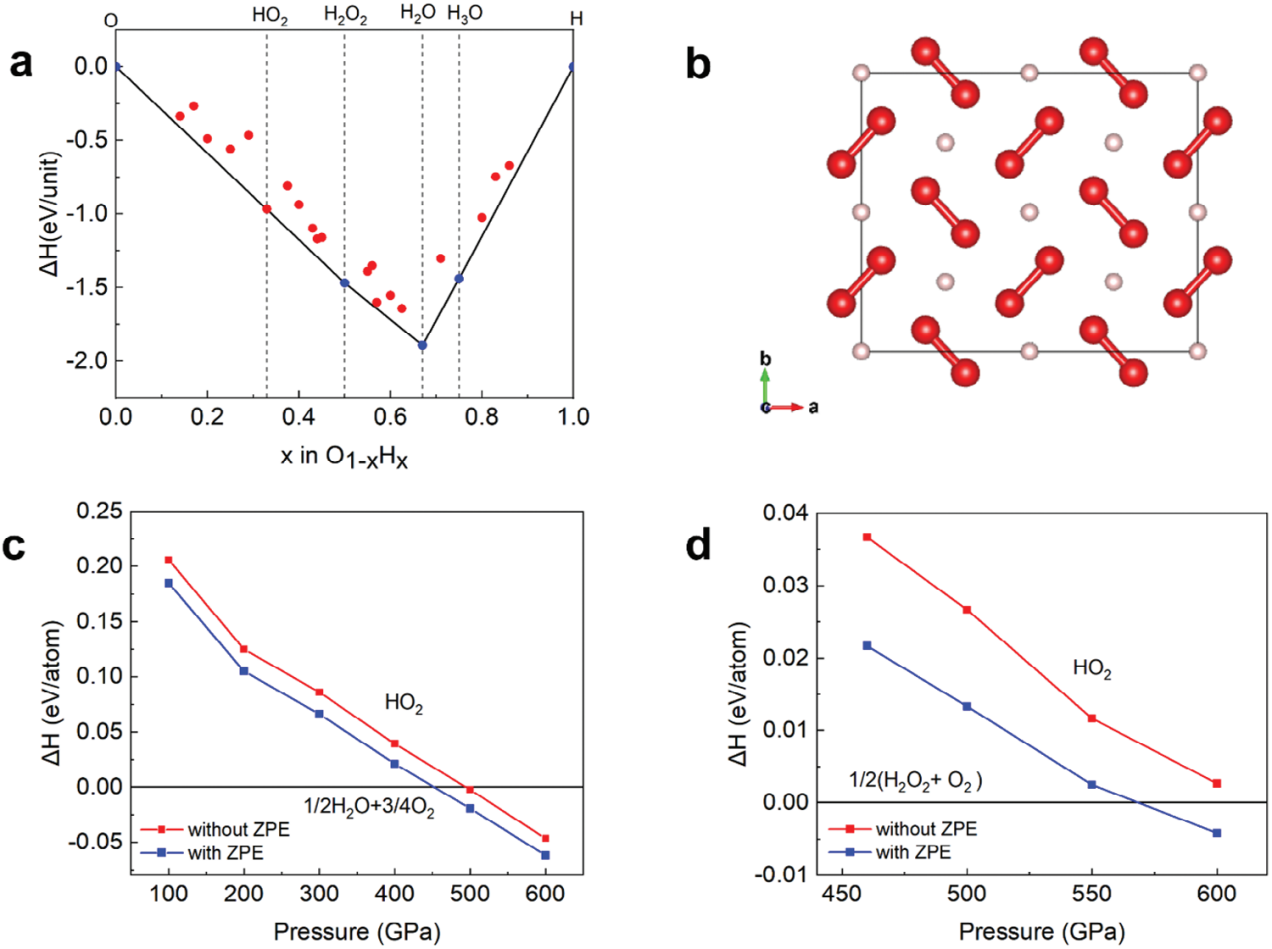
Formation enthalpy and crystal structure of HO2. a) Convex hull of formation enthalpy at 500 GPa. The energetically stable structures (exactly lie at the convex hull) are represented by blue circles, the others are represented by red circles. b) The crystal structure of HO_2_ at 500 GPa. The pink and red spheres correspond to H and O atoms, respectively. c) The formation enthalpy of HO_2_ at 100–600 GPa with and without ZPE contribution referenced by the decomposition line into H_2_O + O_2_. d) The formation enthalpy of HO_2_ at 100–600 GPa with and without ZPE contribution referenced by the decomposition line into H_2_O_2_ + O_2_.

We performed structure predictions on HO_2_ under 100, 200, and 500 GPa using the Ab Initio Random Structure Searching algorithm by generating ≈30000 structures for various compositions.^[^ ^15^ ^]^ The results tell that the ground state at zero temperature with standard Density Functional Theory (DFT) is ice, agreeing with previous literatures and experiments. However, we also found that with pressure, HO_2_ comes to close the thermodynamically stable line and at 200 GPa, the difference in formation enthalpy is quite small, merely ≈0.02 eV per atom (Figure S1, Supporting Information). Thus this structure may become stable as the pressure increases further. The convex hull at 500 GPa shows that HO_2_ is much closer to the convex hull and almost lies at the line (**Figure** **1**a). The deviation from the convex hull line is decreased to around 0.002 eV per atom. The crystal structure is shown in Figure 1b. As we can see, it possesses the equivalent structure motif as LiO_2_.^[^ ^10^ ^]^ The HO_2_ structure is with an orthorhombic space group *Pnnm* and the lattice parameters are: a = 3.43 Å, b = 2.84 Å, c = 1.78 Å at 500 GPa. H localizes at 2a (−1.5, −1.5, −0.5) and O localizes at 4 g (−0.62, −0.85, 0.5) sites.

The thermodynamic stability of HO_2_ relative to its decomposition into H₂O and O₂ has been investigated through enthalpy calculations within the pressure range of 100–500 GPa. Our findings indicate that at pressures ≈500 GPa, HO_2_ becomes more stable than its decomposition products, as illustrated in Figure 1c. This suggests that HO_2_ energetically stabilizes at pressures exceeding 500 GPa. Minor variations in results were observed with different pseudopotentials, as depicted in Figure S2 (Supporting Information), with Local Density Approximation (LDA) functional results showing a decrease in the onset pressure by ≈100 GPa compared to Perdew‐Burke‐Ernzerhof (PBE) results. Additionally, the inclusion of the zero‐point energy (ZPE) term, based on the expectation that ice has a higher ZPE than HO₂, reduced the stability pressure of HO₂ by ≈50 GPa. Considering the longstanding assumption that H₂O is the most stable compound in the simple O‐H binary system, this finding is unexpected.

H_2_O_2_ is another important compound in the H‐O system. It is noted that Zhang et al. predicted a high‐pressure H_2_O_2_ phase with the *Pbca* space group under round 423–600 GPa,^[^ ^16^ ^]^ which is also cross‐checked by our structure predictions. In order to investigate the thermodynamic stability of HO_2_ against the predicted H_2_O_2_ phase, we also calculated the formation enthalpy under 460–600 GPa, following the reaction: 1/2 (H_2_O_2_ + O_2_) → HO_2_. It is indicated that including ZPE correction, HO_2_ becomes more stable than the H_2_O_2_ phase at ≈600 GPa, as illustrated in Figure 1d.

Phonon calculations of HO_2_ were performed under different pressures to check its dynamic stability. It turns out that HO_2_ is dynamically stable in the pressure range of 70–1000 GPa. The detailed phonon spectrums are shown in Figure S3 (Supporting Information). There are no imaginary phonon modes in the above pressure range and instability due to imaginary phonon modes occurs ≈60 GPa.

### 2.2 Electronic Properties

We examined the electronic structures of HO_2_ under 70–500 GPa. Significantly, there exist some discrepancies between different theoretical methods for superoxides. Previous computational results on LiO_2_, NaO_2,_ and KO_2_ unveil that DFT calculations using the generalized gradient approximation (GGA) method results in that they are metallic,^[^ ^5^, ^17^ ^]^ while the others using Heyd–Scuseria–Emzerhof (HSE) hybrid functional predict insulating behaviors with wide band gaps.^[^ ^18^ ^]^ This is because, the inherent self‐interaction errors of the typical DFT functionals such as PBE in GGA approximation will always lead to the excessive delocalization of electrons, which is not so accurate as the picture of electronic structure given by HSE method.^[^ ^19^ ^]^ Plus, the GGA‐PBE approximation cannot give a reasonable bond length for dimer structures.

**Figure 2:**
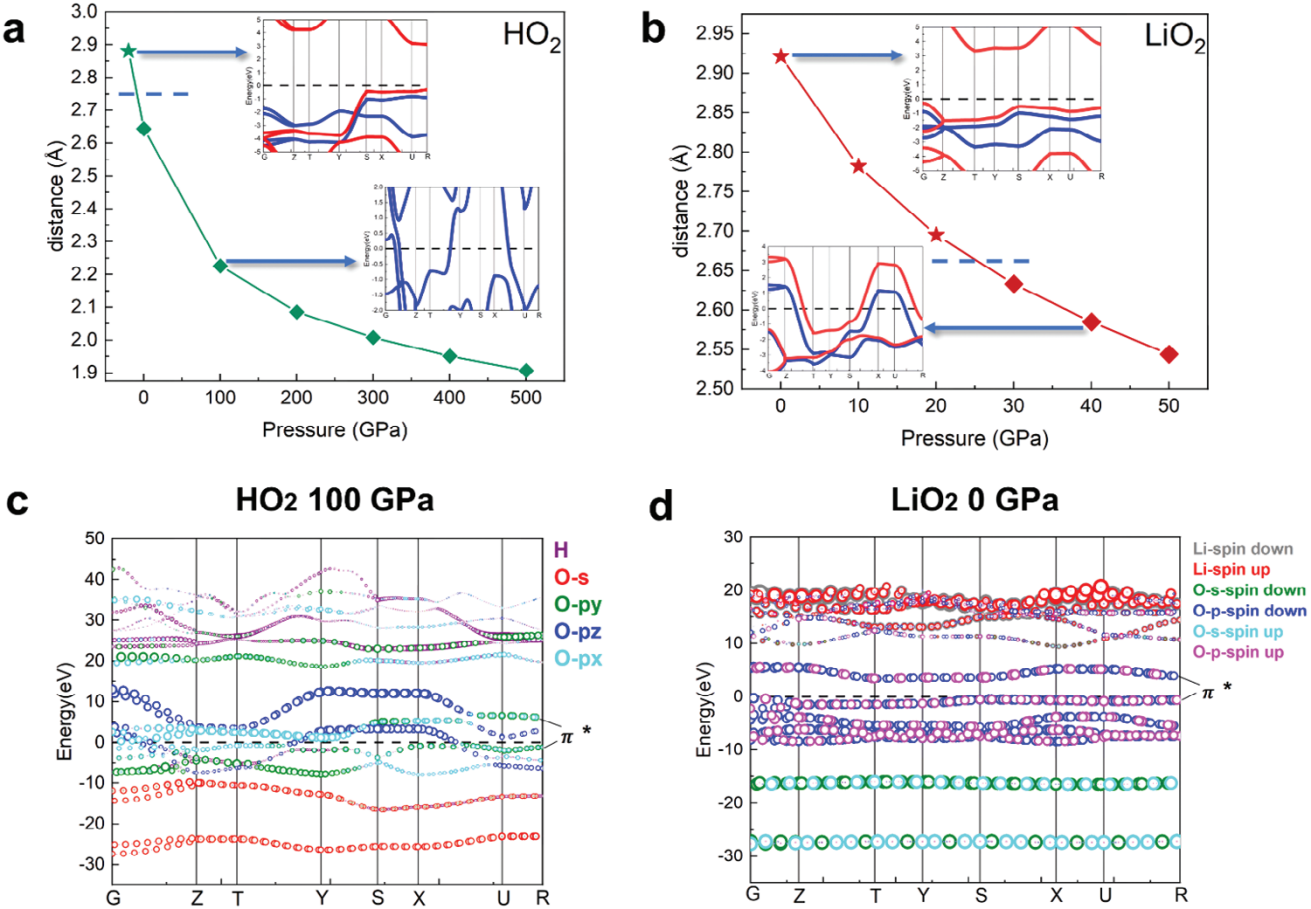
Analysis of electronic structures of HO_2_ and LiO_2_. a,b) Distances between adjacent O_2_^−^ on HO_2_ a) and LiO_2_ b) under different pressures. The stars represent insulators and the diamonds represent metals. The insets in a,b) are the band structures of HO_2_ and LiO_2_ under corresponding conditions by HSE calculation, respectively. c,d) The projected band structures of LiO_2_ at 0 GPa and HO_2_ c) and 100 GPa d).

The electronic band structures of HO_2_ were calculated using the HSE method. Our results indicate that HO_2_ remains metallic across the pressure range of 70–500 GPa, a behavior that is markedly different from other superoxides, such as LiO_2_.^[^ ^18^ ^]^ While HO_2_ exhibits metallic properties, LiO_2_ is an insulator under ambient conditions. To investigate the origin of this disparity and provide a comparison with other superoxides, we also calculated the band structures of LiO_2_ in its stable pressure range (0–50 GPa). Although LiO_2_ shows insulating behavior with a wide bandgap of approximately 3.6 eV under ambient pressure—consistent with previously reported data^[^ ^18^ ^]^—it transitions to a metallic state under high pressure due to the overlap of pressure‐induced 𝜋^*^ orbitals, signifying a Wilson transition. This transition is clearly observable in the band structures before and after the pressure application, as illustrated in **Figure** **2**a,b. Detailed band structures at various pressures are provided in Figures S4, S5 (Supporting Information).

As discussed earlier, all O_2_⁻ possess doubly degenerate 𝜋^*^ orbitals, with one being occupied and the other unoccupied. If the overlap of the unoccupied 𝜋^*^ orbitals between adjacent anions is sufficiently large, metallic behavior emerges.^[^ ^20^ ^]^ Conversely, insufficient overlap of the 𝜋^*^ orbitals can trigger the Jahn–Teller effect, which reduces the system's symmetry, creating a gap between the 𝜋^*^ orbitals and leading to a pressure‐induced insulator‐metal transition. The energy level diagram of 2p molecular orbitals of O_2_^−^ is illustrated in Figure S6 (Supporting Information), where we can see the splitting of 𝜋^*^ states more clearly. Specifically, the projected band structures and the associated Density of States (DOS) of HO_2_ and LiO_2_ (Figure 2c,d, and Figure S7, Supporting Information) confirm that the electrons near the Fermi level in both compounds originate from 𝜋^*^ orbitals. With two electrons occupying each band, along with the energy level of 2p orbitals of O_2_^−^, we can easily know which bands correspond to 𝜋^*^ states. Additionally, a comparison of the distances between adjacent O_2_⁻ anions in HO_2_ and LiO_2_ reveals that the distance in HO_2_ is considerably smaller (Figure 2a,b), facilitating greater orbital overlap and contributing to its metallic nature. To further substantiate this, we applied negative pressure to HO_2_, creating a hypothetical structure with increased distances between adjacent anions. The resulting structure exhibited insulating behavior with a bandgap of ≈3.4 eV, despite the phonon instability, thereby indicating that HO_2_ also undergoes an insulator‐metal transition under these conditions.

Overall, our findings on the electronic structures demonstrate the following: 1) HO_2_ exhibits metallic behavior, and 2) the overlap of 𝜋^*^ orbitals can induce an insulator‐metal transition in superoxides, with pressure serving as a driving force for this overlap. These insights provide a valuable framework for designing energy storage systems, such as superoxide‐based alkali metal‐O_2_ batteries, and help explain the persistent discrepancies in the metallic or insulating properties observed in different superoxides. Furthermore, our results highlight that pressure can be effectively utilized to modulate the band gap of these superoxides, offering a potential pathway for tuning their electronic properties.

### 2.3 Superionic Behavior

**Figure 3:**
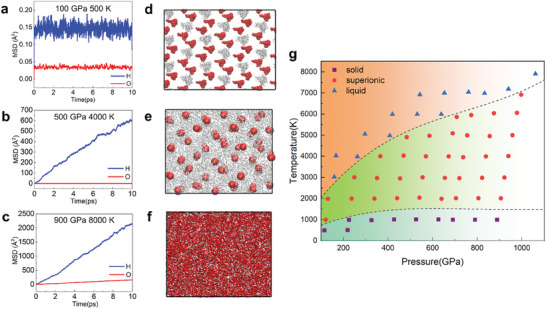
The dynamical properties of HO2 by AIMD simulations. a–c) The mean square displacement (MSD) of HO_2_ under corresponding conditions. d–f) The snapshots of MD trajectories. The white and red spheres represent H and O atoms respectively. g) The phase diagram of HO_2_. The purple squares, red circles, and blue triangles represent solid states, superionic states, and liquid states, respectively.

We performed ab initio molecular dynamics (AIMD) simulations on HO_2_ under high‐pressure and high‐temperature conditions. **Figure** **3**a–f presents the averaged mean squared displacement (MSD) and snapshots of the AIMD trajectories, respectively. The AIMD results reveal that at low temperatures, the MSD values for all hydrogen and oxygen atoms remain nearly constant and close to zero, indicating that the atoms are confined near their equilibrium positions—characteristic of a solid state. As the temperature increases, the MSD values for oxygen remain unchanged, while those for hydrogen begin to increase linearly with time, signifying that hydrogen atoms leave their original positions and diffuse freely within the sublattice formed by solid oxygen atoms. This behavior is indicative of the superionic state, a unique phase where the material exhibits properties of both solid and liquid simultaneously.^[^ ^12d–f^ ^]^ The superionic state is commonly observed in compounds containing light elements under extreme conditions and holds significant importance in functional materials and planetary science. At higher temperatures, both oxygen and hydrogen exhibit diffusion, signaling the melting of the structure and the transition into a liquid phase.

To get a comprehensive understanding of the dynamical properties of HO_2_, we constructed a systematic P‐T phase diagram (Figure 3g). The phase diagram depicts reasonable solid, superionic, and liquid regions. It can help us to identify the specific states of HO_2_ in certain pressure and temperature conditions. It is noteworthy that HO_2_ is in a dynamically stable region when 100 GPa < P < 500 GPa and it is in both energetically and dynamically stable regions when P > 500 GPa in this phase diagram. Although HO_2_ is not energetically favorable under 100–500 GPa, it may exist if there is an excess of oxygen. The investigation of HO_2_ in 100–500 GPa also can help us better understand its properties toward ambient conditions. Moreover, pressure can affect superionic transition temperature and melting point. As we can see in the phase diagram, HO_2_ structures under higher pressures have wide superionic regions, which means higher melting points and superionic temperatures.

As discussed previously, the superionic state of HO_2_ is characterized by high mobility and rapid diffusion of hydrogen atoms. To quantify this, we derived the diffusion coefficients from the AIMD simulations and applied the Nernst–Einstein equation to calculate the ionic electrical conductivities of HO₂ across various pressures and temperatures. As shown in Figure S9 (Supporting Information), hydrogen diffusion increases with rising temperature, transitioning from the solid state to the superionic phase, and eventually to the liquid phase, as reflected in both diffusion coefficients and ionic electrical conductivities. Under higher pressures, HO_2_ exhibits lower diffusion coefficients and reduced ionic electrical conductivities. Specifically, the ionic electrical conductivities in superionic HO_2_ are calculated to be 27.02–71.58 (Ω cm)⁻¹ at 100 GPa, 15.89–71.43 (Ω cm)⁻¹ at 500 GPa, and 11.31–69.54 (Ω cm)⁻¹ at 900 GPa. These values are lower than those of H_2_O due to the lower hydrogen content but significantly higher than those of LiO_2_, owing to the lightness of hydrogen.^[^ ^12^, ^21^ ^]^

### 2.4 Li‐O‐H System

However, based on our phonon calculations, pure HO_2_ is dynamically unstable under ambient pressure, making its synthesis under such conditions currently unfeasible for practical applications. To address this limitation, we propose exploring alternative approaches for creating a hydrogen‐mixed superoxide structure at lower pressures. Given that LiO_2_ is dynamically stable under ambient conditions, one potential strategy involves substituting a few hydrogen atoms for lithium in a large LiO_2_ supercell, thereby creating a hydrogen–lithium mixed superoxide: Li_1‐x_O_2_H_x_ (x < 1). This H‐Li mixed superoxide structure has a high possibility of exhibiting high ionic electrical conductivity of hydrogen and robust stability at relatively low pressures. If this Li‐O‐H system demonstrates dynamical stability at pressures lower than 70 GPa, which is the stability threshold for HO_2_, it could offer a promising pathway for obtaining stable HO₂ in the future. It is also noteworthy that superconductivity has been observed in certain novel Li‐rich Li_m_O compounds,^[^ ^22^ ^]^ suggesting that the hydrogen‐containing Li‐O‐H structure may also exhibit superconducting properties. The specific methodology for constructing the Li‐O‐H structure involves generating several LiO_2_ supercells and systematically replacing some of the lithium atoms with hydrogen. The hydrogen content will significantly affect the dynamical stability, as higher hydrogen concentrations may introduce imaginary phonon frequencies, leading to instability.

**Figure 4:**
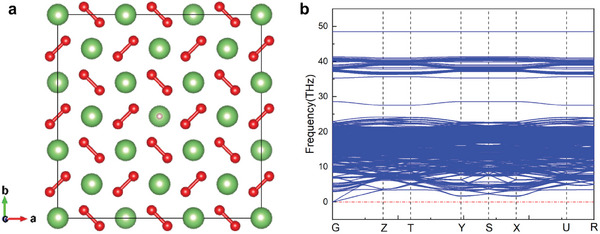
The structure and phonon dispersion of Li53O108H under 60 GPa. a) The crystal structure of Li_53_O_108_H, where the green, white, and red spheres correspond to Li, H, and O atoms. b) The phonon dispersion of Li_53_O_108_H under 60 GPa.

Remarkably, we successfully identified a dynamically stable Li_1‐x_O_2_H_x_ structure: Li_53_O_108_H. Phonon dispersion calculations indicate that this structure remains dynamically stable at 60 GPa (**Figure** **4**), which is lower than the minimum pressure required for the dynamical stability of HO_2_. Future research will focus on strategies to further increase the hydrogen content and reduce the pressure threshold for dynamical stability. This result opens new possibilities and provides inspiration for the design of hydrogen‐lithium mixed superoxides.

## 3 Conclusion

In summary, we have discovered stable proton superoxide HO_2_ under high pressure, exhibiting energetically stability starting from ≈450 GPa. HO_2_ remains metallic throughout its entire stability range, distinguishing it from other alkali‐metal superoxides. Our analysis reveals that its metallic nature originates from the pressure‐induced overlap of 𝜋^*^ orbitals between adjacent O_2_⁻ anions, which also drives insulator‐metal transitions in both HO_2_ and LiO_2_. The interatomic distances between adjacent O_2_⁻ can serve as a key indicator of conductivity in alkali superoxides.

Additionally, HO_2_ exhibits superionic behavior under high pressure and temperature, where the high mobility and diffusion of hydrogen contribute to elevated ionic electrical conductivity. We identified a hydrogen‐containing Li‐O‐H structure (Li_53_O_108_H) dynamically stabilized at lower pressures than pure HO_2_, offering a route to achieve stabilization under reduced conditions.

These findings uncovered the novel proton superoxide HO_2_ at extreme conditions, advancing our understanding of superoxide and superionicity under extreme conditions and enriching the diversity of superoxide compounds and the H‐O system.

## 4 Experimental Section

### Ab Initio Calculations

The ab initio calculations were performed by the Vienna ab initio simulation package (VASP) package with the projector augmented wave (PAW) method.^[^ ^23^ ^]^ The geometry optimizations and enthalpy calculations were conducted by a standard GGA functional approximation in the framework of PBE and the electronic structures were calculated by a hybrid functional method in the framework of HSE (α = 0.48).^[^ ^24^ ^]^ The 2s^2^2p^4^ and 1s^1^ electrons were treated as valence electrons for O and H atoms. The 1000 eV for the plane‐wave energy cutoff was used and an 8 × 8 × 8 mesh within the Monkhorst–Pack scheme for k‐point sampling.^[^ ^25^ ^]^ The convergence threshold of 0.02 eV Å^−1^ in force was selected for the configurations. The phonon and ZPE calculations were performed by PHONOPY code.^[^ ^26^ ^]^ For crystal structure searching, AIRSS was used to search H_x_O_y_ (x = 1–8, y = 1–8) system.^[^ ^27^ ^]^

### Molecular Dynamics (MD) Simulations

The AIMD simulations were conducted with NVT ensemble using the Langevin thermostat implemented by VASP.^[^ ^28^ ^]^ 800 eV for plane‐wave energy cutoff was used and gamma point for *k*‐point sampling. The total simulation times were with the total 10 ps and the timestep was set to 0.5 fs because of the fast‐moving protons.

### Electrical Conductivity

The Nernst–Einstein equation (σ = DNq^2^/kBT) was employed to calculate electrical conductivity. In the equation, q is the carrier electric charge (1e for H atoms), D is the carrier diffusion coefficient, N is the carrier density, and T is the temperature. All these values could be extracted from the AIMD trajectories.

## Conflict of Interest

The authors declare no conflict of interest.

## Supporting information



Supporting Information

## Data Availability

The data that support the findings of this study are available in the supplementary material of this article.
