# Supplementary material for: Stability of Proton Superoxide and its Superionic Transition Under High Pressure
Source: Adv Sci (Weinh). 2025 Jan 13;12(9):2415387. doi: 10.1002/advs.202415387 (PMC11884553; doi:10.1002/advs.202415387)
Supplement: Supplementary file 1 — Supporting Information [file ADVS-12-2415387-s001.docx]

Supporting Information

Stability of Proton Superoxide and its Superionic Transition under High Pressure

Zifan Wang, Wenge Yang, and Duck Young Kim*

Supporting Information for

**Stability of Proton Superoxide and its Superionic Transition under High Pressure**

Zifan Wang, Wenge Yang, Duck Young Kim*

Zifan Wang, Wenge Yang and Duck Young Kim

Center for High Pressure Science & Technology Advanced Research (HPSTAR), Shanghai 201203, P.R. China

*E-mail: [duckyoung.kim@hpstar.ac.cn](mailto:duckyoung.kim@hpstar.ac.cn)

Duck Young Kim

Institute for Shanghai Advanced Research in Physical Sciences (SHARPS), Shanghai 201203, P.R. China

**This file includes:**

Figs. S1 to S9


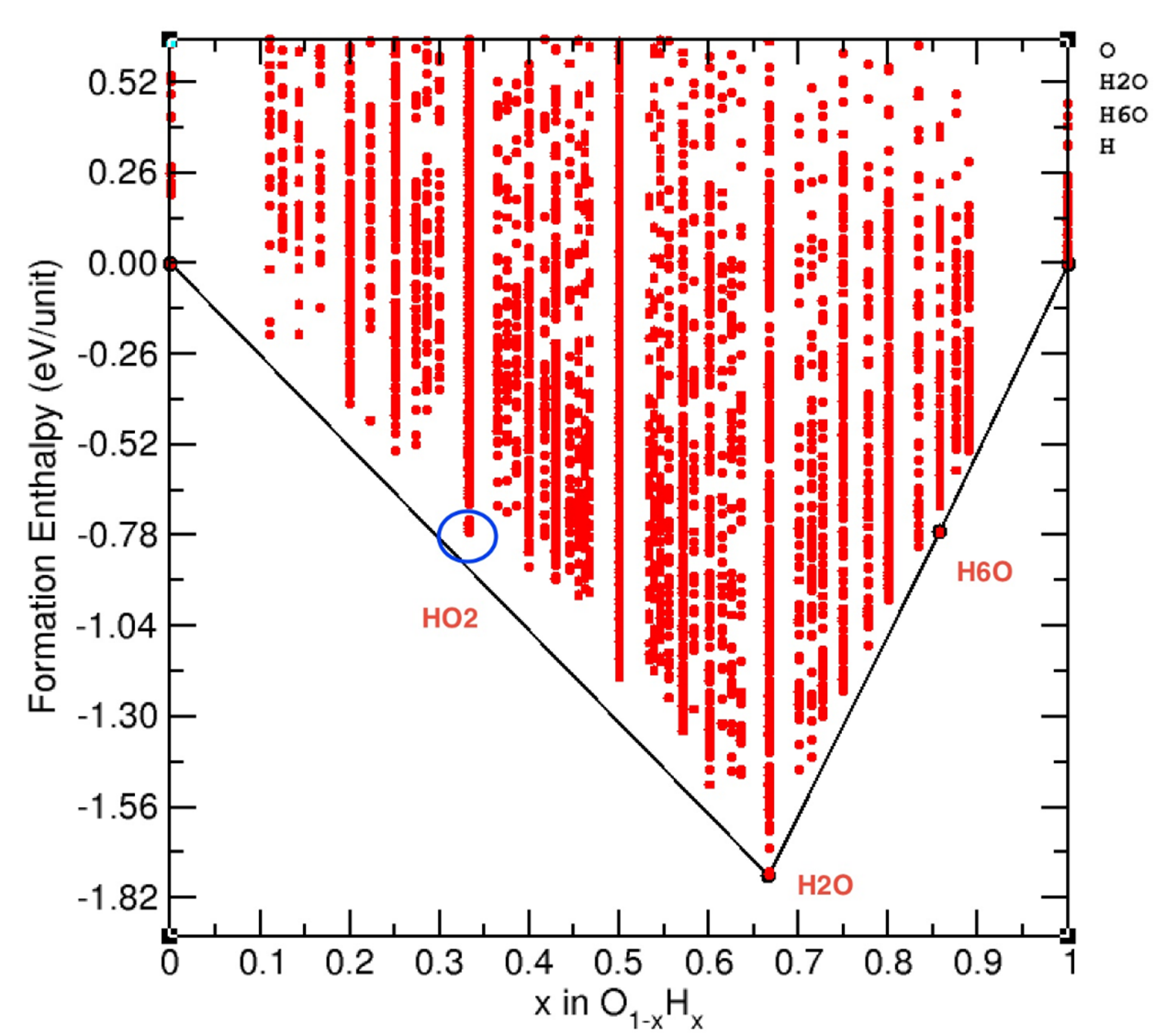


**Fig. S1. The convex hull of formation enthalpy at 200 GPa.**


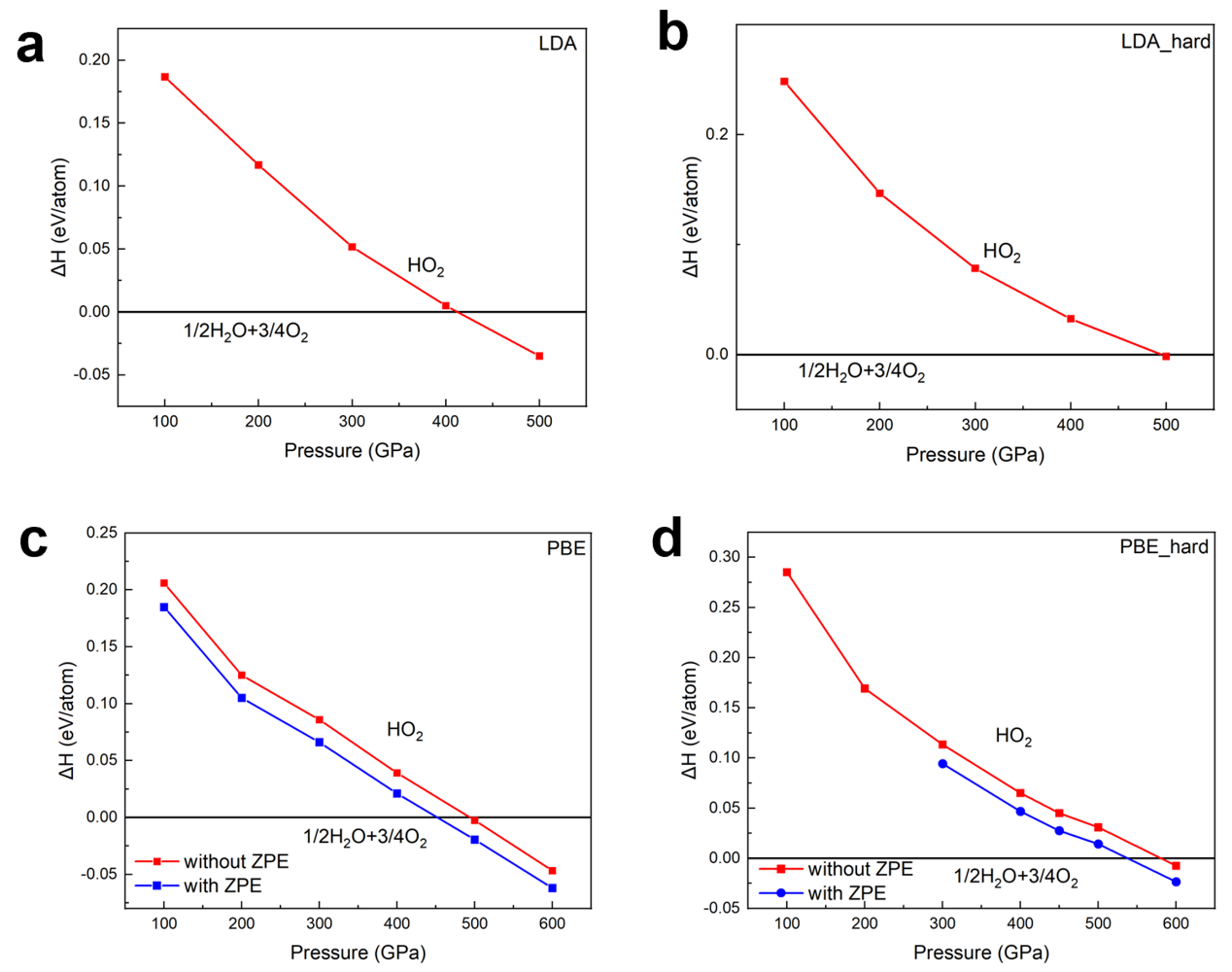


**Fig. S2. The formation enthalpy of HO_2_ under various pseudopotentials. a: LDA, b: LDA_hard, c: PBE, d: PBE_hard.**

**
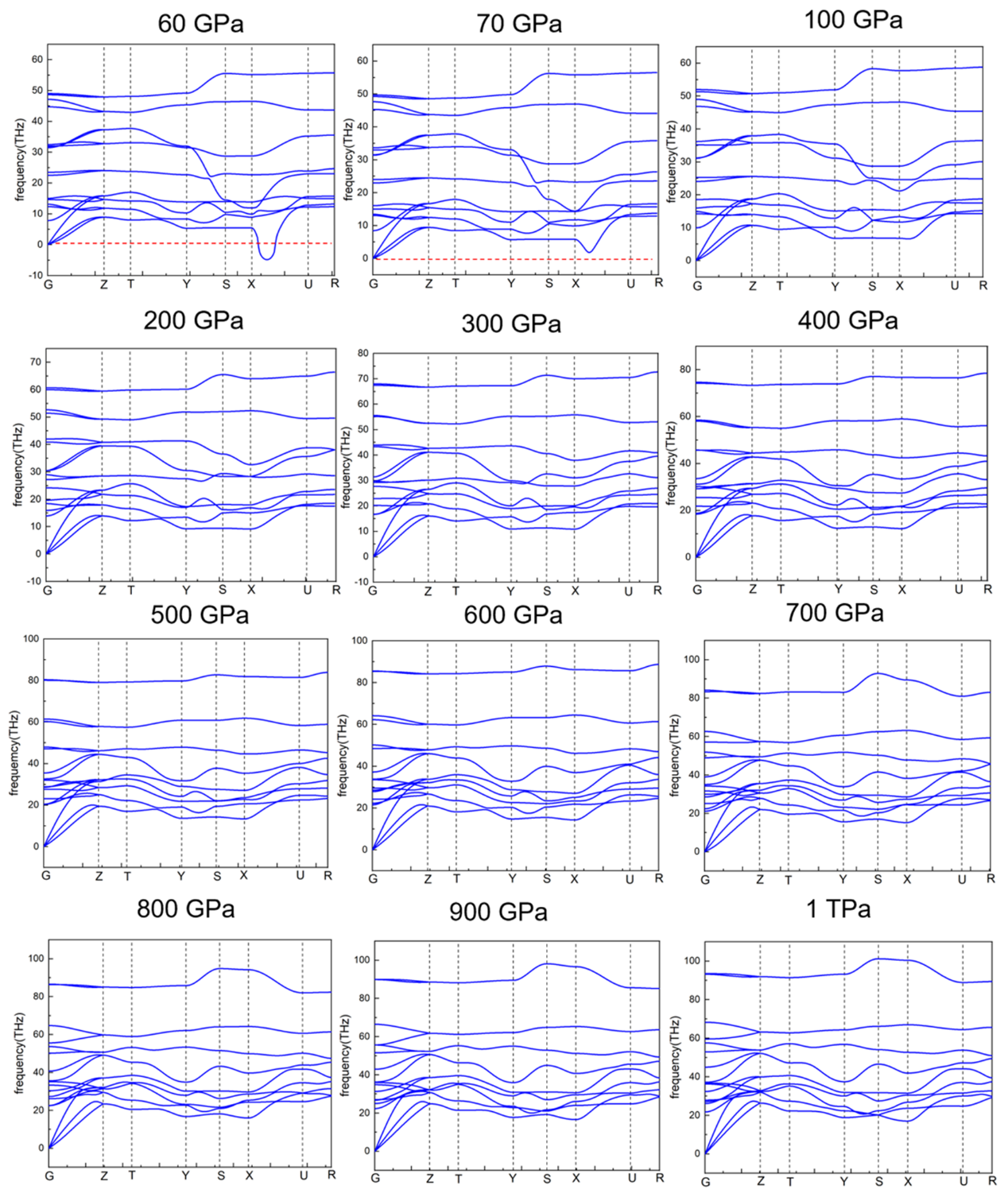
**

**Fig. S3. The phonon dispersions of HO_2_ at 60 GPa -1 TPa.**

**
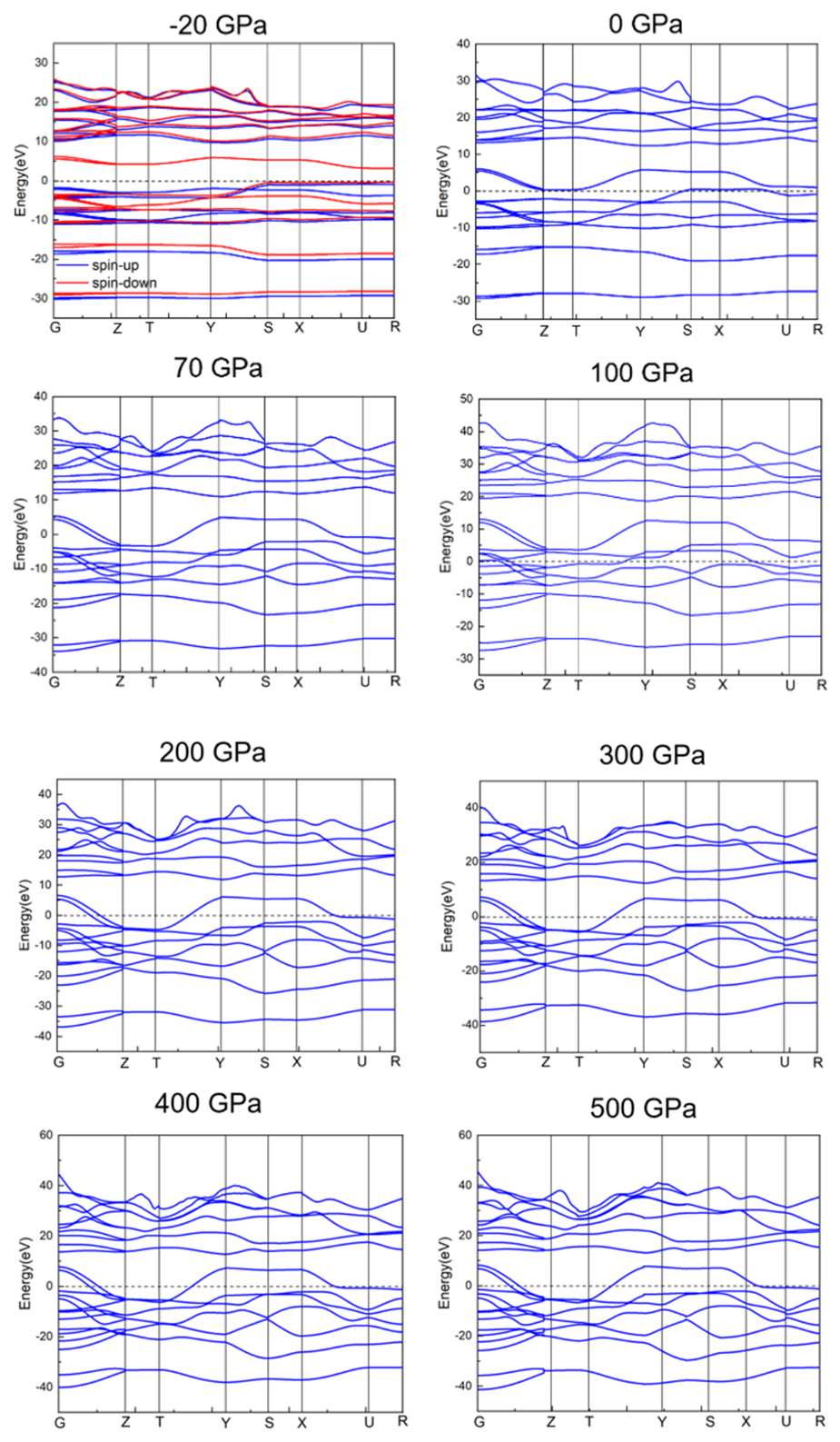
**

**Fig. S4. The band structures of HO_2_ at -20 GPa -500 GPa.**

**
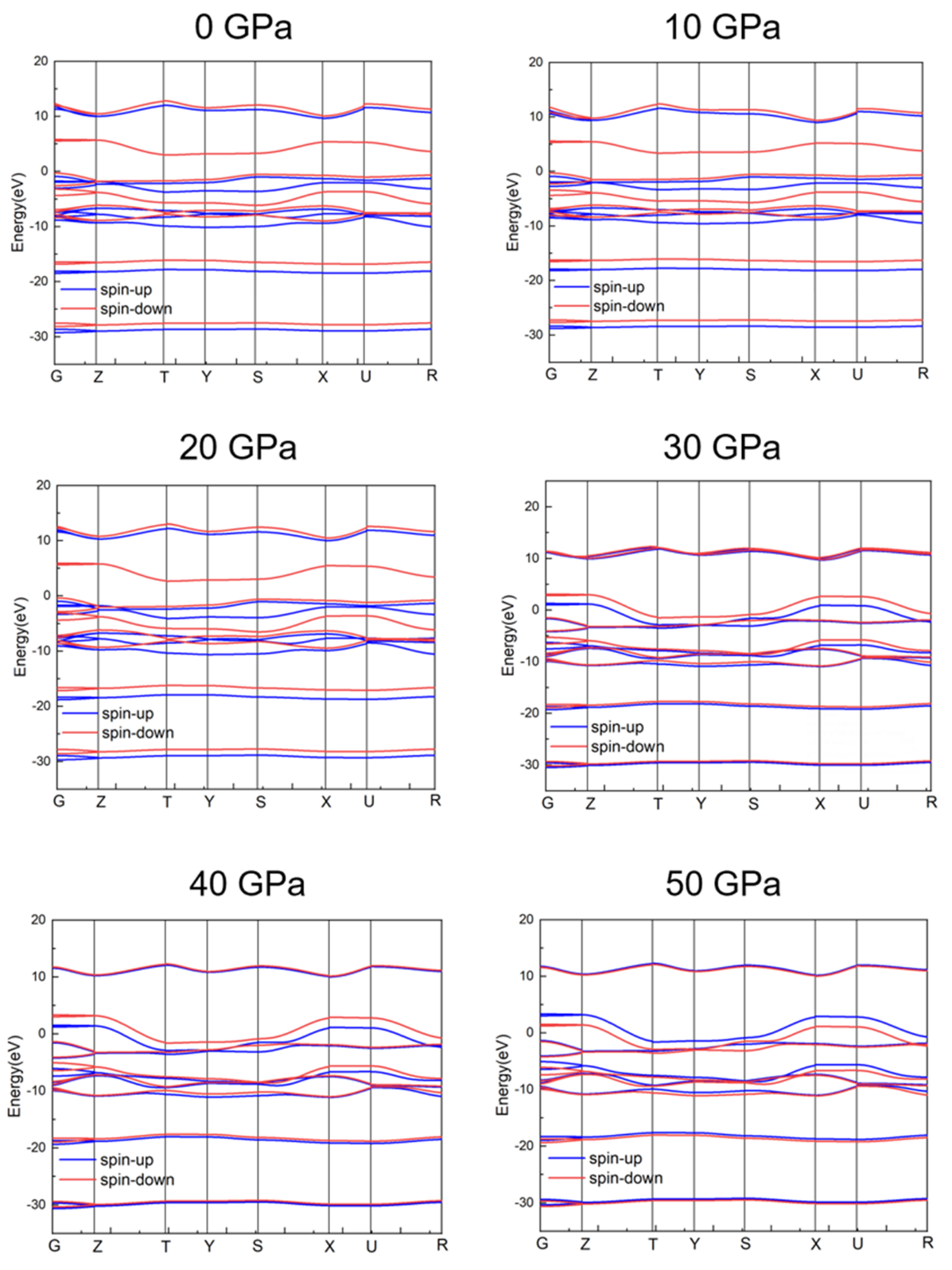
**

**Fig. S5. The band structures of LiO_2_ at 0 GPa -50 GPa.**

**
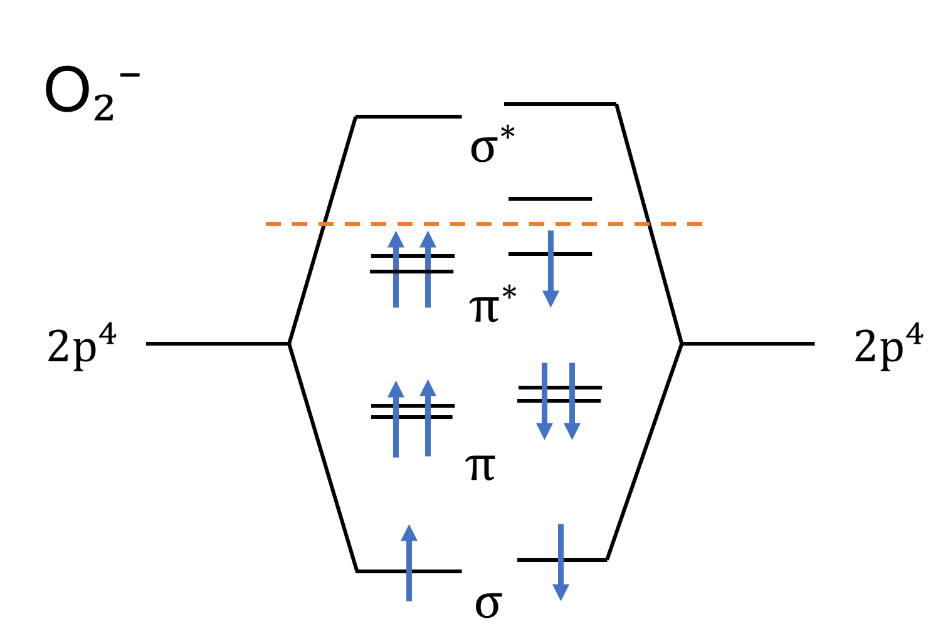
**

**Fig. S6. The energy level diagram for the superoxide O_2_^-^. The red dotted line indicates the separation between the occupied and unoccupied states.**

**
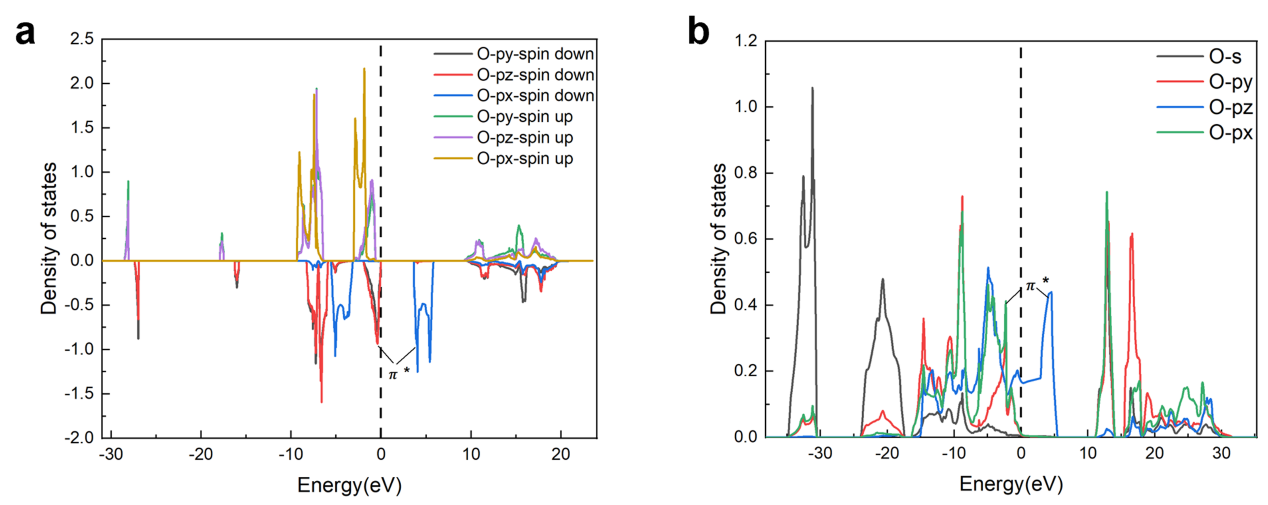
**

**Fig. S7. The projected density of states of LiO_2_ at 0 GPa (a) and HO_2_ and 100 GPa (b).**

**
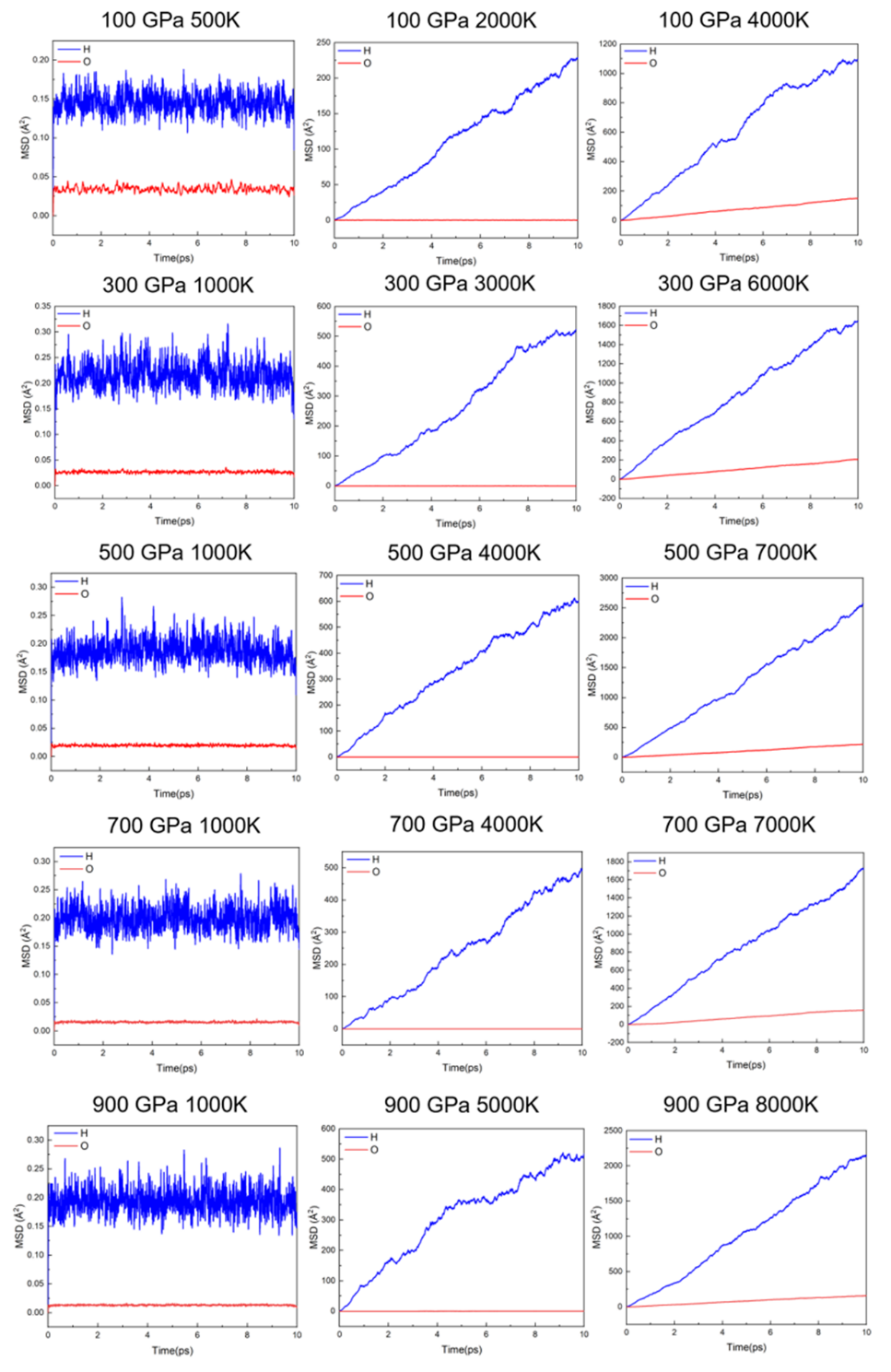
**

**Fig. S8. The mean square displacement (MSD) of HO_2_ under different pressures and temperatures.**

**
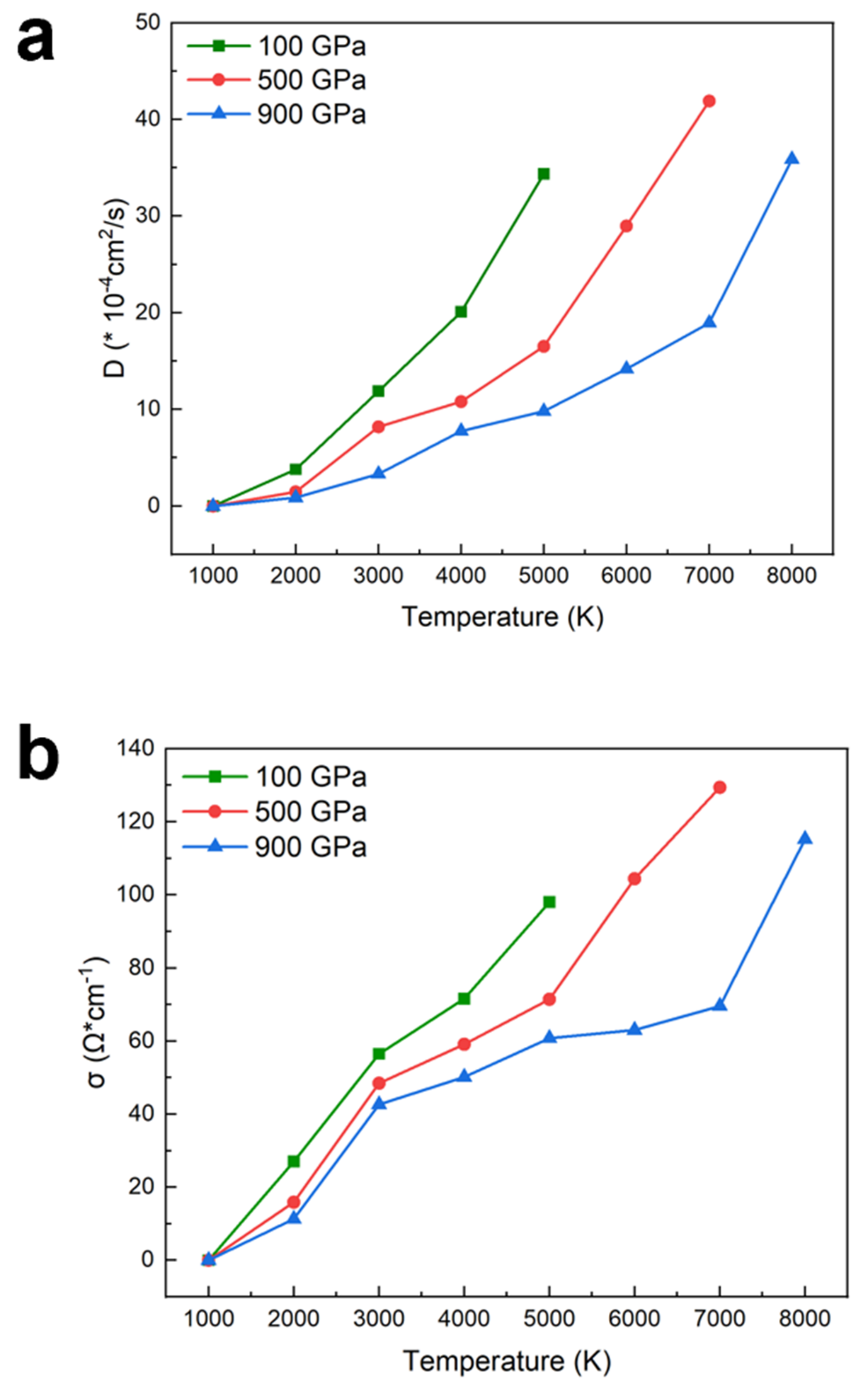
**

**Fig. S9. The diffusion coefficients (a) and ionic electrical conductivities (b) of HO_2_ under different pressures and temperatures.**
